# Foot-and-Mouth Disease in Massachusetts

**Published:** 1903-03

**Authors:** Austin Peters

**Affiliations:** Chief of Cattle Bureau of Massachusetts State Board of Agriculture


### THE JOURNAL

OF

### COMPARATIVE MEDICINE AND VETERINARY ARCHIVES.

VOL. XXIV. *MARCH, 1903.* No. 3.

### FOOT-AND-MOUTH DISEASE IN MASSACHUSETTS.

BY AUSTIN PETERS, M.R.C.V.S.,

CHIEF OF CATTLE BUREAU OF MASSACHUSETTS STATE BOARD OF AGRICULTURE.

THE most unexpected occurrence for the year, and one that has made a great deal of work in a short space of time, has been the outbreak of foot-and-mouth disease.

Foot-and-mouth disease is well-known on the continent of Europe, and has also caused great ravages among the live-stock in England. In the last few years, however, it has been practically eradicated from England, several years having passed without an outbreak in that country, until a year or so ago, when it was introduced again in some way from the continent; but the damage caused by its last introduction has been limited. In European countries, however, it is a source of great loss to the farmers.

In Germany it is known as *Maul und Klauenseuche;* in France, as *fievre aphtheuse ;* in Italy it is known as *afta epizootica*, and in England, besides being spoken of as foot-and-mouth disease, it is also known as epizoötic aphtha.

It is a disease peculiar to cloven-footed domestic animals—the ox, sheep, goat and swine; but very rarely may be communicated by them to the horse, dog and cat; and it is said even occasionally to infect poultry, and also man. It occurs also among wild ruminants, such as deer, buffalo, camel, giraffe, and antelope.

The disease is characterized by vesicular eruption on the mucous membrane of the mouth and surface and edges of the tongue ; it also appears in the interdigital space, and around the top of the foot at the juncture of the hair and horn. The vesicles break, leaving superficial ulcers.

The disease is accompanied in the early stages by fever, which is particularly high just before the eruption of the vesicles appears. It is highly infectious, being disseminated, not only by the cohabitation of sick with healthy animals, but also by manure, litter, stable utensils, clothing and boots of attendants and veterinary surgeons, and also by cattle cars. Even driving healthy cattle over roads previously traversed by cattle suffering from the disease may be sufficient to produce it in the susceptible animals.

Milch cows suffering from foot-and-mouth disease also have a vesicular eruption upon the udder and teats, and in such cases the milk becomes a medium of infection. After two or three days the epithelium, which is raised by the vesicles on the udder or teat, peels off. It may peel so as to leave the entire teat raw, making it difficult to milk the animal and also leading to an extension of the inflammation to the inside of the teat and to the udder, ruining one or more quarters. This complication seems to have been quite frequent during the recent outbreak.

The foot-and-mouth disease has undoubtedly existed for centuries, probably for the last two thousand years. It seems to be a native of Western Asia and Eastern Europe, and has long been known in India and upon the steppes of Russia. As commerce between civilized nations increased and means of communication developed, it spread over Europe from the east to the west, until now it prevails from the Caspian Sea to the Atlantic Ocean. It also exists in India, Ceylon, Burmah, and the Straits Settlements. With the development of literature its description became more accurate, and accounts of its spread over Europe in the seventeenth and eighteenth centuries, show it to have prevailed extensively in Italy, Germany and France. Toward the end of the eighteenth and early in the nineteenth century it reached Western Europe, but did not gain access into England until 1839, and was not known in Denmark until 1841.

Foot-and-mouth disease seems to be a native of localities in Eastern Europe or Western Asia, and an exotic in some other countries. For example, it was imported into Canada in 1870, being introduced by two Shorthorn cows brought from Liverpool, and appeared in localities in New York State and some of the New England States; but it does not seem to have assumed a severe form, and in a few months entirely disappeared. Its behavior in thus disappearing may be partly due to its having been introduced in the autumn, and as cattle in the North are housed during the winter months, there is very little communication between herds; but it may also in part be due to its exotic character. The last outbreak in the United States was in the early eighties (about 1884), when some cattle imported from England were landed at Portland, Me., and driven to the United States quarantine station a short distance away. Soon after a yoke of oxen was driven over the same road, which later developed foot-and-mouth disease—the imported cattle having it when landed—and gave it to the owner's herd; but the disease was soon suppressed, and there has been no reappearance of it until the past autumn.

The United States Bureau of Animal Industry of the Department of Agriculture spares no pains to protect the live-stock interests of the country from contagious animal diseases; hence it is unlikely that this malady will ever secure a foothold here, owing to the stringent quarantine regulations imposed by the Federal Government. Cattle, sheep, other ruminants and swine can be brought into the United States only at points designated by the Department of Agriculture, the ports of entry on the Atlantic seaboard being only at Boston, New York and Baltimore, near which places quarantine stations have been established. All neat cattle are held at these stations for ninety days, and all sheep and swine for fifteen days after landing. The reason for holding cattle longer is to guard against contagious pleuropneumonia; the period for which the sheep and swine are held is sufficient to guard against all danger from foot-and-mouth disease. Neat cattle also have to be found free from tuberculosis as determined by the tuberculin test before being allowed to go beyond the quarantine station limits. Separate gates and lanes are provided for entrance and exit; if, therefore, any contagious disease should make its appearance, there would be no danger of healthy animals becoming infected by being driven over roads where sick animals had previously been.

Importers of animals from England and Europe must also obtain permits from the Department of Agriculture, and, furthermore, must have affidavits that animals come from localities free from contagious disease, and do not pass through infected districts in transit. The collector of the port of entry is notified when the animals arrive, and immediately turns them over to the care of the agent of the Bureau of Animal Industry having charge of the quarantine station.

In countries where foot-and-mouth disease has prevailed as an epizootic, the losses occasioned by it have been very great to the farmer from a pecuniary standpoint; not that the mortality is very large, as only from 1 to 3 per cent. of the infected animals die, but owing to the emaciation due to the fever, and the inability to walk about and eat. This loss is especially heavy when cattle or sheep are being fed for the shambles. Animals nearly ready for the butcher, in a few days lose all the flesh that has taken them months to put on.

Neat cattle have the severest lesions in the mouth, as a rule, and have great difficulty in eating, although lameness may also be present; while among sheep the foot lesions are usually the more severe and they will not walk about in search of food, but spend most of their time lying down.

The period of development from the time of exposure is short, usually from three to eight days; but it may not appear in rare instances, it is said, for from two to three weeks after exposure.

When there are no complications, such as sloughing of the feet as seen in very severe cases, the disease in cattle, sheep, and swine runs a course of from ten to twenty days.

In other animals the symptoms are somewhat the same, viz., vesicles followed by ulcerations either on the feet or in the mouth; but other domestic animals do not seem to be very susceptible. The horse shows lesions in the mouth, but only as a result of licking infected cattle or drinking or eating from buckets or troughs which diseased creatures have used. Dogs, cats and rabbits may have the disease, but it is said to be infrequent, and, in the dog and cat, is the result of their being kept in the stable with infected ruminants or swine, or of their drinking milk from cows with udder lesions.

Foot-and-mouth disease may occur in the human family. Among adults, attendants of cattle, veterinary surgeons, butchers and drovers are most exposed. It may be acquired from diseased animals, and occasionally it is possible for one person to infect another. Among infants it may be caused by drinking uncooked milk from cows having the vesicles of epizoötic aphtha on the udder; it is also said that fresh butter, fresh cheese and whey can convey the germs of the disease,

The period of incubation is quite short, especially when conveyed by milk, in which case it is only from twelve to twenty-four hours before symptoms appear.

In infants the disease is not infrequently fatal. The symptoms consist of fever, headache or dizziness, conjunctivitis, an eruption on the face or around the mouth, and vesicles in the mouth, and among milkers there may be sores on the tips of the fingers around the finger nails (there may even be sloughing of the nails), or in the spaces between the fingers. When vesicles develop in the pharynx they cause great difficulty in swallowing; there may also be vomiting and diarrhœa when the infection takes place through milk, and in the case of infants and small children the disease may then end in death. Recovery usually takes place in ten or twelve days. Among animals it is said that one attack secures only temporary immunity from another. Meat from animals suffering with epizoötic aphtha has never been found to be infectious.

*Etiology*. Foot-and-mouth disease is undoubtedly a germ disease —both clinical and experimental evidence indicate this—but the specific organism which produces it remains to be discovered. There are other diseases which we believe to be due to a microscopic organism of some description—such, for example, as rabies, smallpox, and cowpox—in which the organism is so minute or so difficult to stain or cultivate that it has hitherto not been demonstrated, and the bacterium of epizoötic aphtha is unquestionably one of this character. Nosotti, Klein, Schottelius and Kurth have studied micro-organisms found in connection with the lesions of foot-and-mouth disease, but it does not seem that the true specific cause has yet been discovered.

The vitality of the virus is not considered to be usually very great. Walley says that as a rule the danger ceases at the end of thirty days after the recovery of the last animal, but instances are given in which troughs, hayracks and stables have caused fresh outbreaks after the lapse of several months. Therefore, in taking steps for the eradication of the disease, through disinfection of stables and utensils, with destruction of all litter and manure, cannot be too strictly enforced.

The post-mortem conditions found in animals suffering from foot-and-mouth disease—in addition to the lesions in the mouth and around the feet, and eruptions on the udder previously mentioned—consist of an inflammation of the stomach and intestines, with perhaps erosions of these organs. Especially is this the case in infants, pigs, and calves infected by the use of milk from diseased cows.

The medicinal care of foot-and-mouth disease consists in treating the symptoms as they occur by the use of antiseptic washes, and by the application of dusting powders to the ulcerations. The food should be soft and of an easily-digested character. Large flocks of sheep, where it is impossible to attend to each individual case, are sometimes treated when the foot lesions predominate by driving the entire flock daily through a long wooden trough containing some antiseptic drying powder.[1]

[1] A part of the above is taken from an article written by me recently for the "Reference Handbook of the Medical Sciences."

My attention was first called to the possible existence of foot-and-mouth disease in this section on Wednesday, November 12th. Mr. Dennen, the agent of the Cattle Bureau in charge of the stock-yards at Brighton, came to the office after market on that date and informed me that Mr. Henry S. Turner, one of the Rhode Island Cattle Commissioners, who lives at Scituate, R. I., told him that he was afraid that they had foot-and-mouth disease in Rhode Island. He said that the disease was taken down there by a cow that was brought to the Brighton market from Charlton, in Worcester county. She was taken from there to Chelsea, and in two or three weeks from Chelsea back to Brighton, and from Brighton she went with others to Cumberland, R. I., and that the disease had spread to several herds in that locality.

I immediately wrote to Dr. D. E. Salmon, Chief of the Bureau of Animal Industry at Washington, of what I had heard, and told him I would investigate the matter further and report as soon as I could learn more.

An agent of the Cattle Bureau, Dr. A. W. Draper, happened to be in the office at the time Mr. Dennen told me of Mr. Turner's fears. He was going to Wrentham the next day and to North Attleborough the following day, and I told him to find out what he could when he was down next to the Rhode Island line and report to me. From Dr. Draper's reports of what he was able to hear, on Saturday, November 15th, I went to Wrentham, and with the Inspector of Animals, Dr. Elisha Brastow, drove to Cumberland Hill and saw a herd of cattle owned by H. H. Sprague. Mr. Sprague informed me that the disease was first brought to his place about the first of October by three cows that came from a neighbor to be slaughtered. This neighbor was the man who originally had the diseased cow which came from Brighton. In about ten days after the animals were sent to Mr. Sprague's his herd of thirty-three cattle developed foot-and-mouth disease. He also had five pigs which he told me had it. The animals had evidently had it very severely, as it was a month after the first appearance of the disease when I first saw them, and they had not all fully recovered then. I was not positive as to its being foot-and-mouth disease, and asked where I could see some fresh cases. Mr. Sprague told me of a place in Smithfield, R. I., where the animals had just become infected, and I went home with the intention of going to Smithfield Monday, November 17th; but after my arrival home I was called up by Dr. Edward Knobel, Inspector of Animals in Dedham, who asked me if we had foot-and-mouth disease in this country. I told him that we were not supposed to, as the United States was thought to be free of it; but as he was about leaving the telephone I asked him his reason for asking the question. He said, if we did he thought we had it in a herd of cattle in Dedham. I told him that stranger things had happened, and I would come out there the next day. I drove to Dedham Sunday afternoon, November 16th, accompanied by Dr. Theobald Smith, Professor of Comparative Pathology at Harvard University, whom I invited to go with me. We went to Mr. John K. Burgess' place in Dedham, where we found thirty-eight cows and a bull, all of which had been sick for about a week. The history there was that about three weeks before he bought a cow from a Brighton dealer and kept her about a week. He did not like her because she did not eat well and was lame, and returned her to the dealer. In about ten days after returning this cow his cattle were all taken sick. Driving home, I asked Dr. Smith what his opinion was, and he said that if it was not foot-and-mouth disease he did not see what else it could be, and after discussing the matter we decided that it was my duty to telegraph Dr. Salmon at Washington at once. I accordingly telegraphed Dr. Salmon on the evening of November 16th, saying: "I believe we have foot-and-mouth disease in Rhode Island and Massachusetts. Come at once or send representative." The result of the telegram was the appearance, November 19th, of Dr. John R. Mohler, one of Dr. Salmon's agents. By that time I began to hear of other herds, and was able to show Dr. Mohler cases in Sharon. Dr. Mohler's reports were evidently disquieting enough to lead to other experts being sent to confirm my diagnosis. On November 25th Dr. Leonard Pearson, State Veterinarian of Pennsylvania, and Dean of the Veterinary Department of the University of Pennsylvania, appeared, and the following day, November 26th, Professor James Law, of Cornell University, Ithaca, N. Y., arrived as special agent of the United States Bureau of Animal Industry. They saw cases in Dedham, Needham, Chelsea, Revere, Everett, and Concord, and were satisfied that the trouble was foot-and-mouth disease. Dr. Salmon came to Boston Tuesday, December 2d, as the result of their confirmation of the diagnosis, and since then the State and Federal authorities have been working in co-operation.

At a conference between the Governor of Massachusetts and the Chief of the Bureau of Animal Industry of the United States, Department of Agriculture, and the Chief of the Cattle Bureau of the Massachusetts State Board of Agriculture, at the State House, December 4th, it was agreed that if the United States Department of Agriculture would pay 70 per cent. of the value of animals that were killed because they were infected with foot-and-mouth disease, or had been exposed to it, the appraisal to be based on what the animals were worth when in a state of health, and made by an expert in the values of cattle, who was also to be a citizen of Massachusetts, the State would authorize the slaughter of infected or exposed animals where the public good required it.

On December 5th, therefore, at a meeting of the Governor and Council, the following resolutions and order were adopted:

COMMONWEALTH OF MASSACHUSETTS,
COUNCIL CHAMBER,
December 5, 1902.

WHEREAS, The foot-and-mouth disease, declared to be a contagious disease by the laws of this Commonwealth, exists to an alarming degree among the cattle, sheep, and swine of the Commonwealth; and

WHEREAS, In the opinion of the Chief of the Cattle Bureau of the State Board of Agriculture the public good requires the destruction of certain cattle, sheep, and swine which have been exposed to said contagious disease; and

WHEREAS, The Chief of the Bureau of Animal Industry of the United States Department of Agriculture, by authority of the Secretary of Agriculture, has agreed to reimburse the owners of animals destroyed in accordance with law by payment to the owners of 70 per cent. of the appraised value of such animals, such appraisal to be made by an expert in the value of cattle, who shall be a citizen of the Commonwealth and appointed by said Chief of the United States Bureau of Animal Industry, and to be based upon the value of such animals when in a state of health;

Now, therefore, it is hereby ordered that the following order of the Chief of the Cattle Bureau of the State Board of Agriculture be approved.

Adopted in Council, December 5, 1902.

E. F. HAMLIN,
Executive Secretary.

BOSTON, December 5, 1902.

*To all Persons Whom it May Concern:*

By virtue of the power and authority vested by law in the Cattle Bureau of the State Board of Agriculture under the provisions of Chapter 90 of the Revised Laws and Chapter 116 of the acts of 1902, you are hereby notified that foot-and-mouth disease, which is a contagious disease and is so recognized by the laws of this Commonwealth, exists among cattle, sheep, and swine in some sections of this State.

You are hereby further notified that in order to prevent the spread of this disease this bureau has issued the following order:

It is hereby ordered that all cattle, sheep, or swine which have foot-and-mouth disease, or which have been exposed to it, shall be killed in those cases where, in the opinion of the Chief of the Cattle Bureau, the public interests require it.

This order shall take effect upon its approval by the Governor and Council.

Approved in Council, December 5, 1902.

AUSTIN PETERS,
Chief of Cattle Bureau.

E. F. HAMLIN,
Executive Secretary.

Agents of the United States Bureau of Animal Industry were appointed as agents of the Cattle Bureau of the Massachusetts State Board of Agriculture in order to give them the necessary authority to carry out measures required for stamping out the disease.

The slaughter of diseased and exposed animals for stamping out foot-and-mouth disease is an unusually radical way of dealing with this malady, and the only excuse for it is on account of the financial and commercial interests involved. The United States Secretary of Agriculture issued an order December 27th forbidding the export of cattle, sheep and other ruminants or swine from the port of Boston until further orders. As large numbers of cattle and sheep were shipped from this port, the loss to the railroad and steamship companies has been very heavy. It is estimated that it has cost Boston $100,000 a day in loss of business; furthermore, the presence of the disease in New England is a menace to the live-stock interests of the country in case it should spread to other States, and for this reason more vigorous measures have been adopted for its eradication than would otherwise be warranted.

It is not usually a fatal disease. Very many animals recover and are restored to their original value. If the premises where the diseased animals are can be kept under quarantine until the last case recovers, and the buildings and materials contained in them have been thoroughly disinfected, that usually seems to be sufficient to stay the ravages of this plague; but in order to expedite matters for the sake of allowing New England ports to be opened for the export of animals again, and for the purpose of being able to declare this country free of foot-and-mouth disease in order that Great

Britain will allow our animals to be landed at her wharves again, these very radical steps have been taken.

The United States Bureau of Animal Industry disinfects premises where animals are killed, and where they have recovered and the United States has not yet decided to kill them, the Cattle Bureau of the State Board of Agriculture disinfects the premises.

Immediately upon ascertaining the presence of foot-and-mouth disease in the community, the following letter was sent to every inspector of animals in Massachusetts:

COMMONWEALTH OF MASSACHUSETTS,
CATTLE BUREAU OF THE STATE BOARD OF AGRICULTURE,
ROOM 138, STATE HOUSE,
BOSTON, November 17, 1902.

*To the Inspector of Animals:*

DEAR SIR: A disease similar to foot-and-mouth disease has been lately called to my attention; in fact, the symptoms are so much like those of foot-and-mouth disease I think it will very likely turn out to be that malady. The cattle are first noticed to drool, then have blisters appearing in the mouth or on the tongue, which later break, forming little ulcerating sores. They do not eat well while their mouths are sore. They also have blisters appearing around the feet, especially in the space between the two divisions of the hoof, and in some instances blisters appear on the udder, which afterward break and form raw sores on the udders or teats.

The disease may also be communicated to sheep and swine. I wish, if you meet any cases of this kind, you would quarantine the cattle, sheep, or swine that have the disease or that have been exposed to it, forbidding the owner to take them off his premises or to drive them across or on the public highway. If his fields have not any public highway between the barn and the lots he wishes to turn them into, he can turn them out if he wishes, provided they do not come in contact with his neighbors' cattle; otherwise they are to be kept in the stable.

When you quarantine any such animals you can call the disease on your quarantine notice "foot-and-mouth disease," giving the owner the original copy of quarantine notice and sending duplicate here. As this trouble may prove to be serious and important, I wish you to give it your careful attention, and carry out any orders directed to you in connection with it faithfully. It may also be conveyed by the shoes and sometimes by the clothing. I think if you have any herds to deal with where the trouble exists, you had better wear rubbers and an old waterproof coat that can afterward be sponged off with some disinfectant solution before going among other cattle.

Yours truly,

AUSTIN PETERS,
Chief of Cattle Bureau.

In addition to these letters, inspectors of animals in towns adjoining the Rhode Island line had the following letter sent to them in order to stop, as far as possible, the moving of cattle from Rhode Island into Massachusetts:

COMMONWEALTH OF MASSACHUSETTS,
CATTLE BUREAU OF THE STATE BOARD OF AGRICULTURE,
STATE HOUSE.
BOSTON, November 18, 1902.

DEAR SIR: I think that the rules and regulations concerning bringing cattle into Massachusetts have not been observed very well lately, and I wish to call your attention to the fact that persons bringing cattle into Massachusetts must have a permit, and that the cattle must be tested with tuberculin at their expense and risk—except calves under six months old or beeves for immediate slaughter—by a veterinarian satisfactory to me. I recognize the fact that a good many cattle brought in from Rhode Island are cattle that have been sold through the Brighton market, and that as cattle from Brighton, from out of the State, are tested at the Brighton yards, of course no further test is necessary.

I write just now, as I consider this is a particularly important matter, as I am informed that there seems to be a disease among cattle in Cumberland, R. I., which appears to be of a contagious character, the cattle having sore mouths, sore feet, and sores on the udders, and I wish the inspectors of animals in the bordering towns to keep a lookout for cattle coming in from Rhode Island, and if any cattle are brought in from without the State they are to quarantine them and send duplicates to me; or if any cattle present symptoms of sore mouths, sore feet, or have any sores on the udders, they are not only to quarantine them, but to quarantine the herd of the person buying any such animals until I can investigate the matter and see that there is no danger of the spread of the disease.

If you quarantine any cattle, sheep, or swine, you can make the notice of quarantine read "foot-and-mouth disease."

Yours truly,

AUSTIN PETERS,
Chief of Cattle Bureau.

(To be continued.)

